# Cost awareness amongst irish ophthalmologists

**DOI:** 10.1007/s11845-023-03332-7

**Published:** 2023-04-29

**Authors:** Barry Power

**Affiliations:** 1 Royal Victoria Eye and Ear Hospital, Dublin 2, Ireland; 2 Ophthalmology Department, Royal Victoria Eye and Ear Hospital, Adelaide Rd, Dublin 2, Ireland

**Keywords:** Costs, Healthcare Economics, Ophthalmology, Sustainability

## Abstract

**Background:**

Healthcare systems have increasingly limited and stretched budgets. Clinicians have a key role in budget allocation. Awareness of the costs of high-use clinical items is important.

**Aims:**

Assess awareness of the cost of commonly utilised clinical items amongst Irish Ophthalmologists

**Methods:**

Irish ophthalmologists were contacted and asked to fill out an anonymous survey. We assessed knowledge of hospital costs of surgical materials, medications and anti VEGF drugs as well as retail pharmacy costs of commonly prescribed medications. The cost of items to the hospital was recorded from pharmacy and ward order receipts from a single university hospital. The costs of items to the patient were calculated by taking an average of 3 prices charged by local retail pharmacies. For each estimate we calculated the absolute error from the true price. We calculated the mean absolute errors (MAE) and percentage errors (MAPE) across the different groups.

**Results:**

We received responses from 47 participants (15 Senior House Officers, 11 Registrars, 21 Consultant/Community Ophthalmologists). Despite 70% of respondents agreeing that the cost of an item should have a major role in its use, the average estimate was 124% inaccurate. Less than 50% of responses were within 50% of the true cost of the item. Self-perceived knowledge was acknowledged to be limited or very limited in 73% of responses.

**Conclusions:**

We demonstrate variable and limited levels of cost awareness. Seniority and better self-perceived knowledge were not found to be associated with better estimate accuracy.

## Background

Healthcare expenditure grows year on year. The estimated 2020 health expenditure in Ireland is €20.3 billion [1]. Clinicians are responsible for 70% of budget expenditure in healthcare [2]. Pharmaceutical expenditure ranges from 8.5% to 29.6% of health spending within Organisation for Economic Co-operation and Development countries and is increasing faster than other areas of health-care spending in almost all countries [3].

High-value, cost-conscious care refers to care that aims to assess the benefits, harms, and costs of interventions and consequently to provide care that adds value [4]. Given the fact that we all work within a limited budget, the value of a drug or item cannot be appreciated without knowing the cost. Despite this, many clinicians receive little or no formal training on the costs of the materials they use and the drugs they prescribe. To manage budgets effectively, clinicians must have a reasonable understanding of the costs associated with their practice.

In addition to costs to health systems and departments, it is important to understand how much different drugs cost patients. In some cases, patients continue to purchase drugs at high personal cost when suitable alternatives exist [5]. In others, drugs are discontinued unbeknownst to the doctor due to budgetary constraints [6, 7].

The aim of this study was to gauge the level of knowledge possessed by Irish ophthalmologists about the costs of commonly used drugs and clinical equipment. We were also interested to see if there was an association between seniority and estimate accuracy.

## Methods

All currently practicing Irish ophthalmologists were contacted via email. Respondents were asked for estimates, to the nearest Euro, of the cost of a list of common clinical items. We divided the items into 4 categories – cost of: medications to the hospital (9 items), surgical materials to the hospital (15 items), anti-VEGF medications to the hospital (3 items) and medications to the patient (8 items). Participants were asked to record their level of training as Senior House Officer [SHO], Registrar/ Specialist Registrar [SPR] and Consultant/Community Ophthalmologist (COP).

The cost of each item was per unit (i.e. per bottle of medication, per pair of gloves etc.) and this was stated clearly in each question. Where different volumes are in common use, a specific volume was indicated. We recorded the hospital costs of items from ward and pharmacy receipts. We calculated average costs to the patient by taking an average from three local retail pharmacies.

In our statistical analysis we looked at the 4 categories separately and overall. We also looked at each level of seniority separately. Naturally, some estimates were above and some below the true cost. This limits the usefulness of a simple mean estimate per item. The primary measure of the accuracy of estimates that we used was the mean absolute error (MAE). The absolute error of each estimate per item was calculated:

$$\left(\Delta {\varvec{x}}\right)=\left|{{\varvec{x}}}_{{\varvec{i}}}-\left.{\varvec{x}}\right|\right.$$

x^i^ is the estimate; x is the true value.

The MAE was then calculated per item. The MAE expressed as percentages of the true cost of each is the mean absolute percentage error (MAPE). This facilitates comparison between items of different prices. Standard deviation calculations were also performed for each item.

Respondents also answered questions relating to their self-perceived knowledge of cost of common medications/items and the level of influence the price of an item should have on its use in clinical practice.

## Results

There were 47 respondents to the study (21 Consultant/[COP, 11 SPRs/Registrars and 15 SHOs). Just 4% of respondents reported their self-perceived knowledge as very good or good. Limited or very limited knowledge was reported by 47%. Consultants/COPs were more likely to report good or average knowledge than SPR/Reg and SHO groups (43%, 9% and 0% respectively).

**Fig. 1:**
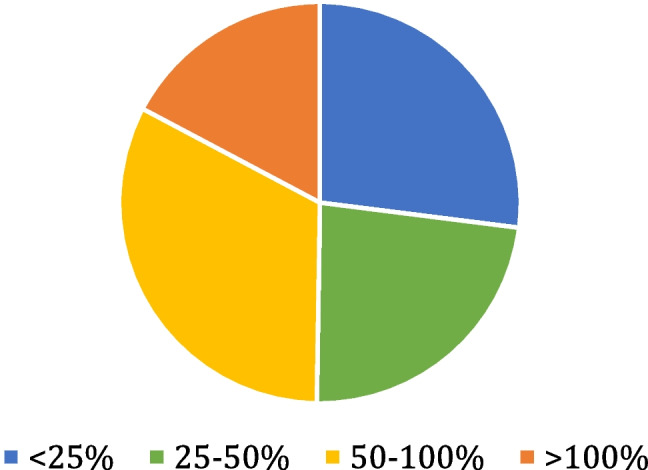
Displays a breakdown the mean average percentage errors of respondents’ estimates

**Fig. 2:**
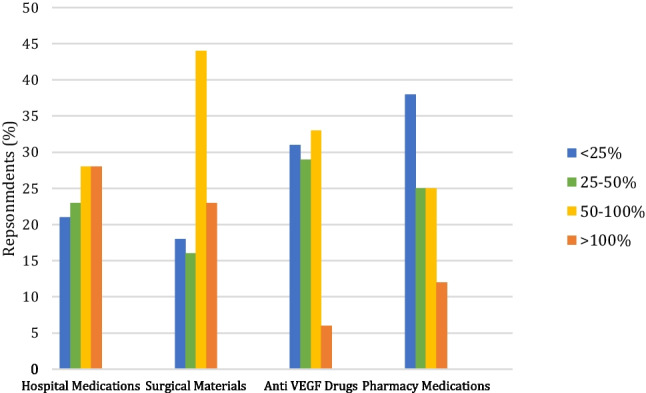
Displays a breakdown of the mean average percentage errors in each of the 4 categories

The majority of respondents agreed that the price of items should factor into their use - 70% responding this should be the case always or as much as patient safety/clinical efficacy allows. Despite this, just 50% of estimates were within 50% of the true cost (Fig. 1). The mean average percentage errors are categorized in Fig. 2.

Estimate accuracy varied widely across different categories of items. The MAPE was highest in the perioperative material section followed by the hospital medications, anti-VEGF drugs and retail pharmacy medications (243, 124, 75 and 53% respectively).

When the respondents were broken down into 3 groups based on seniority, the Consultant/COP group’s estimates were more accurate but there was no statistical difference between the groups (Table 1, P=0.47; Kruskal Wallis). They had the lowest MAPE in 2 of the four groups (hospital medications and perioperative materials) and the lowest overall MAPE.

**Table 1:** Displays the respondents’ estimate MAPEs by seniority

Role	Hospital Medications	Surgical Materials	Anti VEGF Drugs	Pharmacy Medications	Avg
Consultant/COP	103	212	75	64	111
SPR/Reg	134	259	40	67	131
SHO	134	259	109	58	137

Before providing estimates, responders were asked to rate their self-perceived knowledge. No clinicians rated their knowledge as very good, 2 rated it as good with average, limited and very limited selected by 8, 18 and 9 respectively (NR from 10). Self-perceived knowledge was not found to predict better estimates (Table 2, P=0.18, Kruskal Wallis).

**Table 2:** Displays the respondents’ estimate MAPEs by self-perceived knowledge

Rating (N)	Hospital Medications	Surgical Materials	Anti VEGF Drugs	Pharmacy Medications	Avg
Very Good (0)	0	0	0	0	0
Good (2)	81	606	270	40	268
Average (8)	85	139	35	53	92
Limited (18)	114	159	64	62	113
Very limited (9)	137	254	43	60	151

## Discussion

It is clear from the results that the cost of many items are poorly understood by Irish ophthalmologists. This finding has been demonstrated by other studies across a variety of healthcare specialities [8–11]. Allan et al performed a systematic review of 24 studies and found that clinicians consistently overestimated the cost of inexpensive products and underestimated the cost of expensive ones [12]. Doctors estimates were within 25% of the true cost less than one third of the time. We noted similar findings in our cohort (27%).

The hospital cost responses (medications, surgical materials and anti-VEGF drugs) highlighted a significant knowledge gap with less than one third of responses being accurate to +/- 25%. For high cost and frequent use items in particular, this lack of awareness is not conducive with efficient use of a departmental budget. The anti-VEGF medications are a good example of similar drugs with very large price differences. There are a small number of conditions that may respond better to the more expensive Aflibercept and Ranabizumab, but the generic drug Avastin has been shown in multiple trials to be non-inferior in treatment of AMD and retinal vein occlusions associated with macular oedema [13–15]. In a situation where the non-inferiority of a more cost effective option has been proven, it should be first line. Many respondents to our survey were unaware of the extent of the price difference between these agents.

It is alarming that neither seniority nor self-perceived knowledge was associated with more accurate estimates (Tables 1 and 2). The Consultant/COP group had the lowest average error but this difference did not meet statistical significance. Some Community Ophthalmologists (COPs) may have a limited understanding of surgical costs which may explain some of the outliers in this section. That being said, there were poor estimates across all groups, including those who predicted accurate responses. Senior ophthalmologists are department level decision makers and should set the standard for costs awareness amongst other staff.

Clinicians are trained to work up patients, make clinical decisions and formulate treatment plans but these decisions dictate the finances of patients and the health sector as a whole. The dual roles of clinicians and managers are inseparable and increasingly consequential. The judicious use of resources is ultimately the clinicians responsibility.

Improving cost-consciousness amongst doctors is a challenge. Each treatment decision has multiple factors influencing the final outcome. However, with ever increasing healthcare expenditure it is a must. We firmly believe that medical training should include the basics of healthcare economics. Subspecialties of all disciplines should educate their trainees and themselves about the costs of the tests they order and treatments they initiate.
